# Sprague Dawley Rats Show More Severe Bone Loss, Osteophytosis and Inflammation Compared to Wistar Han Rats in a High-Fat, High-Sucrose Diet Model of Joint Damage

**DOI:** 10.3390/ijms23073725

**Published:** 2022-03-28

**Authors:** Kelly Warmink, Jaqueline L. Rios, Devin R. van Valkengoed, Nicoline M. Korthagen, Harrie Weinans

**Affiliations:** 1 Department of Orthopaedics, University Medical Center Utrecht (UMCU), P.O. Box 85500, 3584 GA Utrecht, The Netherlands; j.l.rios-2@umcutrecht.nl (J.L.R.); devinvvalk@gmail.com (D.R.v.V.); n.m.korthagen@uu.nl (N.M.K.); h.h.weinans@umcutrecht.nl (H.W.); 2 Department of Equine Sciences, Utrecht University, P.O. Box 80125, 3584 CS Utrecht, The Netherlands

**Keywords:** animal models, osteoarthritis, osteoporosis, obesity

## Abstract

In animal models, joint degeneration observed in response to obesogenic diet varies in nature and severity. In this study, we compare joint damage in Sprague Dawley and Wistar-Han rats in response to a high-fat, high-sucrose (HFS) diet groove model of osteoarthritis (OA). Wistar Han (*n* = 5) and Sprague Dawley (*n* = 5) rats were fed an HFS diet for 24 weeks. OA was induced 12 weeks after the diet onset by groove surgery in the right knee joint. The left knee served as a control. Outcomes were OARSI histopathology scoring, bone changes by µCT imaging, local (synovial and fat pad) and systemic (blood cytokine) inflammation markers. In both rat strains, the HFS diet resulted in a similar change in metabolic parameters, but only Sprague Dawley rats showed a large, osteoporosis-like decrease in trabecular bone volume. Osteophyte count and local joint inflammation were higher in Sprague Dawley rats. In contrast, cartilage degeneration and systemic inflammatory marker levels were similar between the rat strains. The difference in bone volume loss, osteophytosis and local inflammation suggest that both rat strains show a different joint damage phenotype and could, therefore, potentially represent different OA phenotypes observed in humans.

## 1. Introduction

Osteoarthritis (OA) is a degenerative joint disease that is known to affect multiple tissues in the joint [1]. Although the hallmark of OA is degenerated cartilage with a loss of its mechanical properties and function, the other tissues are also affected. Bone undergoes several changes like the formation of osteophytes, cysts, bone marrow edema and other abnormal (increased) bone remodeling [2,3]. In early OA stages, bone remodeling is fast, with increased bone resorption and subchondral plate thinning, and perforations can be observed [4,5], whereas at later stages, subchondral plate thickening or sclerosis can be observed [3]. In addition, a persistent low-grade inflammation results in changes in the synovium and the infrapatellar fat pad [6]. Inflammatory cells infiltrate the synovium and cause thickening of the synovial lining and the production of inflammatory cytokines [7,8]. In the infrapatellar fat pad, the increased inflammation leads to fibrosis of the adipose tissue [9,10]. All these factors contribute to degeneration of the joint, ultimately leading to chronic pain and functional disability.

Overweight, obesity and associated metabolic syndrome are known risk factors for OA and have been classified as the metabolic OA phenotype. The metabolic OA phenotype has been suggested to be a consequence of increased mechanical loading in weight-bearing joints, systemic metabolic alterations and persistent low-grade inflammation [11]. In addition, systemic alterations associated with metabolic syndrome are thought to aggravate the local low-grade inflammation in the osteoarthritic joint via inflammatory mediators such as adipokines and cytokines, thereby leading to an accelerated OA development [12,13,14,15].

Sprague Dawley and Wistar rats are widely used as both OA and obesity models. Both rat strains are outbred, and although used for similar purposes, they differ in several aspects [16]. Sprague Dawley rats are known to be less physically active and grow at a relatively faster rate than Wistar rats [17], whereas metabolic effects caused by a high-fat diet are reported to be slightly more pronounced in Wistar rats [18]. Another striking difference is that Wistar rats have a much greater longevity. A study that combined data of 3650 healthy control rats reported almost 30% higher mean survival in Wistar rats [19]. This study also found that common spontaneous tumors, such as pituitary tumors and mammary tumors in females, were more prevalent in Sprague Dawley rats. In addition, it is known that the development of osteoporosis after ovariectomy in female Sprague Dawley rats occurs more rapidly and is more severe compared to Wistar rats, indicating there also is a difference in bone characteristics between the two strains [20].

However, little is known about the differences between these strains in OA models. This could be potentially problematic for reproducibility of results, understanding the exact mechanisms that lead to OA, and eventually, therapy development. In previous models, Sprague Dawley rats showed a drastic joint degeneration on a high-fat and high-sucrose diet [21], without any surgical damage trigger, effects that have not been found in other models with Wistar rats [14]. Therefore, in this study, we compare the features of OA in Sprague Dawley and Wistar Han rats in response to a high-fat, high-sucrose (HFS) diet in combination with surgical grooves placed on the femoral condyles to induce OA. We hypothesize based on previous literature that joint damage in response to HFS diet and groove surgery will be worse in the Sprague Dawley rat strain.

## 2. Results

### 2.1. Body Mass and Metabolic Parameters

**Figure 1:**
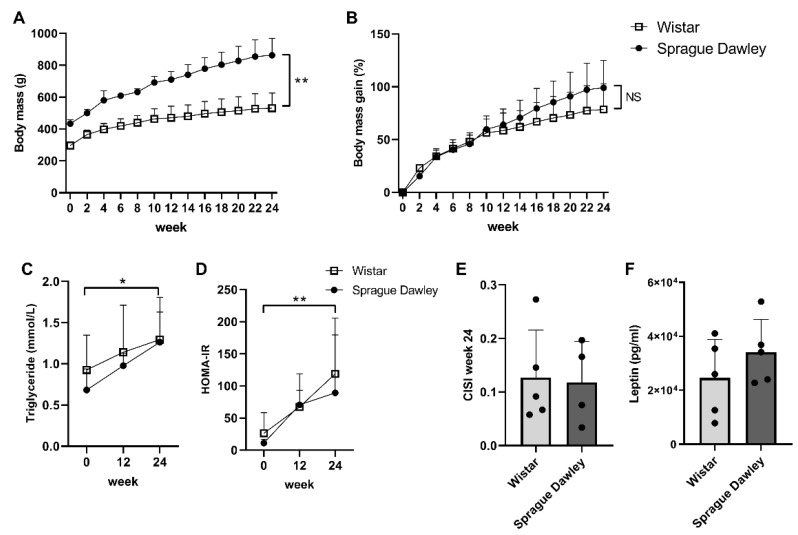
Body mass and metabolic parameters. (**A**) Total body mass over 24 weeks, asterisks indicate *p* < 0.01 at every time point and (**B**) gain in body mass as a percentage of baseline body mass, NS indicates *p* > 0.05 at every time point. (**C**) Plasma triglyceride measurements and (**D**) HOMA-IR determined from fasting glucose and insulin concentrations at weeks 0, 12 and 24. (**E**) CISI score determined from glucose tolerance test and (**F**) serum leptin concentration at week 24. *p* ≤ 0.05 is *, *p* ≤ 0.01 is ** and *p* > 0.05 is not significant (NS).

At baseline, the average body mass of the Sprague Dawley rats was 434 g, whereas the Wistar rats weighed 296.4 g on average (Figure 1A; *p* = 4.1 × 10^−5^). During the 24 study weeks, the Sprague Dawley rats remained heavier than the Wistar rats at all time points (*p* < 0.0001). The body mass gain, expressed as a percentage increase from the baseline body mass, was not significantly different between Wistars and Sprague Dawleys at any time point (Figure 1B; *p* > 0.86). Plasma triglycerides and insulin resistance (HOMA-IR score) increased over the course of 24 weeks for both rat strains (*p* = 0.016 and *p* = 0.0069, respectively); however, there were no differences between Wistars and Sprague Dawleys (Figure 1C,D). In addition, the insulin sensitivity (CISI score) and serum leptin concentration measured in week 24 were similar between the two rat strains (Figure 1E,F). Altogether, these data indicate that the HFS diet had a similar effect on the metabolic status for Sprague Dawleys and Wistars, despite the higher body mass observed in the Sprague Dawley rats.

### 2.2. OARSI Histopathology Score and Inflammation Markers

**Figure 2:**
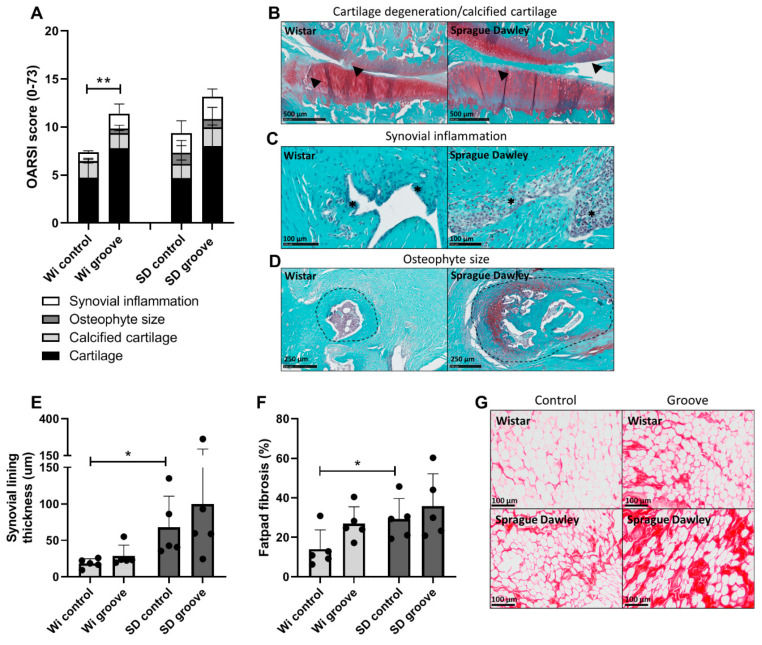
OARSI scores and inflammation. (**A**) Total OARSI score comprised of four OARSI subscores: cartilage degeneration (0–60), calcified cartilage (0–5), osteophyte size (0–4) and synovial inflammation (0–4). (**B**) Example of Saf-O stained knee joint in rats with relative high scores in cartilage degeneration, arrows indicate cartilage damage and GAG loss, whereas the calcified cartilage layer is still intact. (**C**) Saf-O stained images showing high synovial inflammation per group, asterisks indicate the synovial lining. (**D**) Saf-O stained images showing the largest osteophyte that was found per group, encircled by a dashed line. (**E**) Synovial lining thickness measured in µm and (**F**) percentage of infrapatellar fat pad fibrosis as determined by surface area of sirious red staining. (**G**) Example of sirious red staining in the infrapatellar fat pad of Wistar and Sprague Dawley rat, control (**left** knee) and groove (**right** knee) image are from the same animal. *p* ≤ 0.05 is * and *p* ≤ 0.01 is **.

There were no differences between Wistar and Sprague Dawley rats in the total OARSI score (Figure 2A). In the Wistars, the grooved joints showed an increase of 4.1 points in OARSI score compared to the control joints (*p* = 0.025). In Sprague Dawley rats, the average OARSI score was 3.8 points higher in the grooved joints, but here, the difference between grooved and control joint did not reach statistical significance (*p* = 0.087). Cartilage degeneration had the biggest contribution to the total OARSI score (Figure 2A); however, as the groove model is a relatively mild model of OA, we did not see major joint destruction (Figure 2B), and we observed no differences between Wistars and Sprague Dawleys for the individual OARSI subscores (Supplementary Figure S1A–D). There were three animals in the Sprague Dawley group with large osteophytes, resulting in a high spread in this subscore (Figure 2D, Supplementary Figure S1D). Synovial lining thickness and percentage of fibrosis in the infrapatellar fat pad were both increased in Sprague Dawley controls compared to Wistar controls (Figure 2C,E–G; *p* = 0.033 and *p* = 0.045, respectively). Systemic inflammation markers measured in serum showed no difference between the two strains for any of the 27 cytokines and chemokines (Supplementary Figure S2). Local knee inflammation measured as synovial lining thickness and fat pad fibrosis was higher in Sprague Dawley rats, but systemic inflammatory marker levels were similar between the rat strains.

### 2.3. Micro Computed Tomography (μCT) Bone Measurements

**Figure 3:**
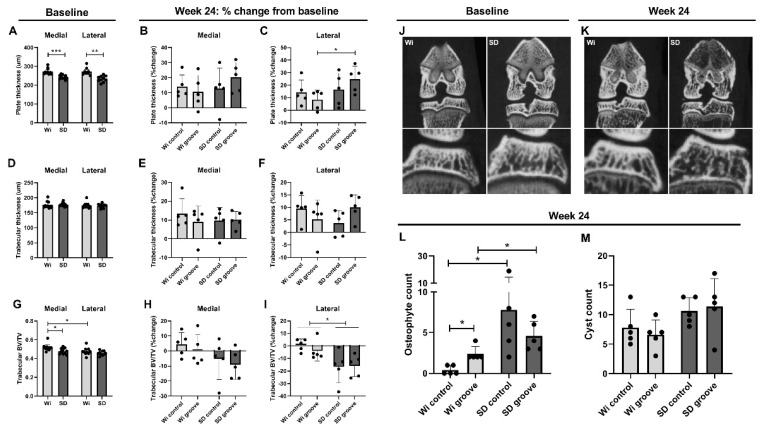
µCT bone measurements, osteophytes and cysts. (**A**) Baseline tibial subchondral plate thickness and (**B**,**C**) percentage change from baseline thickness medial and lateral. (**D**) Baseline tibial trabecular bone thickness and (**E**,**F**) percentage change from baseline thickness medial and lateral. (**G**) Baseline tibial trabecular bone volume fraction and (**H**,**I**) percentage change from baseline volume fraction medial and lateral. (**J**) Baseline µCT images of a Wistar and a Sprague Dawley rat, with close up of the lateral tibia condyle and (**K**) week 24 µCT images of the same Wistar and Sprague Dawley rat at approximately the same coronal plane, lower bone volume fraction in the Sprague Dawley rat can be observed by eye. (**L**) Week 24 µCT osteophyte count and (**M**) cyst count. *p* ≤ 0.05 is *, *p* ≤ 0.01 is **, *p* ≤ 0.001 is ***.

At baseline, tibial subchondral plate thickness was lower in the Sprague Dawleys for both medial and lateral tibial plateau when compared to the Wistar rats (Figure 3A; *p* = 9.0 × 10^−4^ and *p* = 0.002, respectively). Full subchondral plate perforations were not observed. There were some partial perforations, but the number, size and location did not change between baseline and after 24 weeks (results not shown), so these were not considered a result of joint damage in this study. The subchondral plate thickness was increased after 24 weeks for almost all animals in both groups. In the lateral compartment of the grooved joints, the Sprague Dawleys showed an increase in subchondral plate thickness compared to the Wistars (Figure 3C, *p* = 0.021). There was no difference in change in subchondral plate thickness between control and grooved joints for both strains (Figure 3B,C). Tibial trabecular bone thickness was similar between Wistar and Sprague Dawley rats at baseline (Figure 3D), and there were no differences between and within groups after 24 weeks (Figure 3E,F). Tibial trabecular bone volume fraction was lower at baseline in Sprague Dawley compared to Wistar rats on the medial compartment (Figure 3G,J; *p* = 0.048). After 24 weeks, Sprague Dawley rats had a marked decrease in bone volume fraction up to −30%, whereas the Wistar rats had little change from baseline (Figure 3H,I,K). In the lateral compartment, the Sprague Dawleys had significantly lower trabecular bone volume than the Wistar rats for both control and grooved groups (Figure 3I; *p* = 0.022 and *p* = 0.049, respectively). Control or groove treatment did not affect bone volumetric fraction in either rat strain. Absolute values (not corrected for baseline) for subchondral plate and trabecular thickness and bone volumetric fraction at week 24 are shown in Supplementary Figure S3.

The number of cysts and osteophytes were counted on uCT scans performed at 24 weeks. In the Wistar rats, the osteophyte count was elevated in the grooved joints compared to the control joint (Figure 3L; *p* = 0.022). The Sprague Dawley rats showed a much higher number of osteophytes compared to Wistar rats in both control and grooved groups (Figure 3L; *p* = 0.038 and *p* = 0.041, respectively). In contrast to the Wistars, no differences in osteophyte count between control and grooved were observed in the Sprague Dawley rats, suggesting that Sprague Dawley rats are more sensitive to developing osteophytes, which seems to be independent from OA inducing surgery. We observed no significant differences between groups in cyst count (Figure 3M).

## 3. Discussion

In this study, we compared Wistar Han and Sprague Dawley rats in their response to a high-fat, high-sucrose diet combined with surgical groove OA induction. We found that cartilage damage is similar in both rat strains, but that bone changes and local inflammation were substantially worse in Sprague Dawley rats. Overall, Sprague Dawley rats had a thicker synovial lining and a high count of osteophytes. In addition, Sprague Dawley rats had a large decrease in trabecular bone volumetric fraction after 24 weeks compared to baseline measurements. Interestingly, trabecular bone volumetric fraction and subchondral plate thickness were already differing between strains at baseline, indicating that Sprague Dawley and Wistar rats might have a different bone makeup, which could also affect their respective susceptibility for bone changes over time as a result of diet or OA induction. Groove surgery did not seem to affect bone cysts, tibial bone volumetric fraction, subchondral plate thickness, or trabecular bone thickness in both rat strains, so it seems that the differences found in these outcomes are strain-related, rather than a feature of OA induction in this study.

Previous studies showed that female Sprague Dawley rats are more prone to develop osteoporosis than Wistar rats in an ovariectomy model [20]. These results are in line with our observation that Sprague Dawley rats show a marked decrease in bone volumetric fraction over 24 weeks when exposed to a HFS diet, whereas the average bone volumetric fraction in Wistars remains similar to their baseline measurement. In a study by Rios et al., Sprague Dawley rats were fed a HFS diet for 12 weeks without any surgical OA induction. Their most prominent finding on histology was joint damage by the loss of subchondral bone and subsequent collapse of the adjacent cartilage; however, the cartilage itself did not present many signs of degeneration [21]. The loss of subchondral bone was not seen in chow control or intervention groups that received prebiotic fiber supplementation and/or aerobic exercise, indicating the metabolic alterations caused by the HFS diet may play a major role in the effects observed in the subchondral bone metabolism. In our study, the decrease in subchondral bone volume in Sprague Dawley rats did not result in bone collapse. However, the HFS diet used by Rios et al. had a higher sucrose content and a lower fat content than used in this study, which could explain why they observed more dramatic effects in a shorter time period. Overall, it seems that Sprague Dawley rats have a predisposition for bone loss that is not observed in Wistar rats, and we hypothesize that these bone changes are accelerated by a HFS diet and its metabolic effects.

Next to bone changes, Sprague Dawley rats had worse local inflammation as measured by the synovial lining thickness and infrapatellar fat pad fibrosis. Nevertheless, systemic inflammatory factors such as cytokines and chemokines were similar in both strains, suggesting the higher inflammatory levels in Sprague Dawleys are mostly on a local level. This increased inflammation could also be connected to the high number of osteophytes that were observed in Sprague Dawley rats. It is known that local anti-inflammatory treatment with drugs such as triamcinolone acetonide or COX-2 inhibitor celecoxib inhibits osteophyte formation very efficiently [8,22]. Synovial inflammation and, more specifically, the macrophages in the synovial lining are known to play a role in osteophyte formation, which is proposed to occur via TGF-β, BMP2 and BMP4 production by these macrophages [23,24]. Therefore, higher levels of inflammation in the OA joint can contribute to more and/or larger osteophytes.

An important factor to consider when interpreting the results of this study is the difference in body mass between the two strains. Although metabolic parameters and percent increases in body mass were similar, the Sprague Dawley rats did have a higher absolute body mass at baseline, and this may affect the load bearing in the joints. Interestingly, the higher body mass did not lead to more cartilage damage or higher OARSI scores in the Sprague Dawley rats, but it could have affected differences we observed in the bone on µCT (correlations are shown in Supplementary Figure S4). The effects of obesity on bone metabolism are paradoxical; on the one hand, it is thought to increase bone density due to more mechanical loading, but on the other hand, obesity-associated systemic inflammation is related to negative effects on the bone microstructure. At the same time, adipose tissue also produces estrogen and thereby contributes to the prevention of osteoporosis. However, in our study, the higher body mass in Sprague Dawley rats could not prevent the bone loss on volumetric fraction, but the difference in body mass could explain why the Sprague Dawley rats had a larger increase in subchondral plate thickness over 24 weeks to counteract of the heavier loading on the joints.

The results from this study emphasize once again that differences between animal strains need to be considered when designing in vivo animal studies to study bone and joint degeneration. For example, intervention with prebiotic fiber or exercise helps to prevent subchondral bone collapse and joint destruction in the Sprague Dawley HFS model [21], whereas this might not be a successful intervention strategy in Wistar rats as we observe they have no bone loss in response to HFS diet. Hence, we see that different rat models elucidate different aspects of joint degeneration and likely need different treatments to counteract its progression. The complex and multifactorial nature of joint degeneration and the large variation between patients stresses the importance of well-characterized preclinical models to increase understanding of the different phenotypes and eventually find a disease-modifying therapy that is specifically designed for each subtype.

## 4. Materials and Methods

### 4.1. Animals and OA Model

Twelve-week-old male Wistar Han rats (*n* = 5; Crl:WI(Han) Charles-River, Sulzfeld, Germany) and Sprague Dawley rats (*n* = 5; Crl:CD(SD) Charles-River, Sulzfeld, Germany) were fed ad libitum with a high-fat, high-sucrose diet (customized diet “EF HS D12451 mod.”; 20 kcal% protein, 40 kcal% fat of which 94% pork lard, 40 kcal% carbohydrates of which 83% sucrose, Sniff Bio-Services, Soest, the Netherlands), for a period of 24 weeks. Body mass was measured weekly. Twelve weeks after the diet onset, OA was induced by unilateral groove surgery, where local cartilage damage was induced in the right knee joint by making longitudinal grooves on the femoral condyles and trochlea, as described previously [25]. The left knee joints of all animals received no surgical intervention and served as a control. All surgeries were performed under anesthesia (isofluorane (Abbott, Green Oaks, IL, USA)—induced at 4%, maintained at 2.5%) and analgesia (0.01 mg/kg buprenorphine (Schering-Plough, Westerlo, Belgium), injected subcutaneously prior, and 6–8 h after surgery). In week 24 of the experimental protocol, animals were euthanized, and tissues of interest were harvested for further evaluation. Rats were housed per two in open polycarbonate cages (Type IV) under a 12:12 light–dark cycle. This experiment was approved by the Utrecht University Medical Ethical Committee for animal studies (license AVD115002016490) and was in compliance with European Community specifications regarding the use of laboratory animals.

### 4.2. Blood Measurements

At weeks 0, 12 and 24, 16 h fasted blood was obtained from the tail vein and collected in both lithium heparin plasma and clot activator serum tubes (BD Microtainer, BD, Franklin Lakes, NJ, USA). Samples were placed on wet ice and then centrifuged (15 min, 3000 g) within 30 min after collection. Plasma and serum aliquots were stored at −80 °C until analysis. Heparin plasma was used to determine glucose and triglyceride levels (University Veterinary Diagnostic Laboratory of the Utrecht University). Serum insulin levels were determined by ELISA (EZRMI-13K, Millipore, Amsterdam, The Netherlands). Insulin resistance index (HOMA-IR) was calculated according to the equation proposed by Matthews et al. [26], where HOMA-IR = fasting glucose (mmol/L) × fasting insulin (mU/L)/22.5. In addition, a glucose tolerance test was performed at week 24; after 16 h of fasting, the rats were fed 2 g/kg glucose by oral gavage. Blood was collected in a serum tube at 0, 15, 30, 60, 90 and 120 min after gavage from the tail vein for insulin measurement. Blood glucose was measured immediately with a blood glucose meter (OneTouch Verio and Blood Glucose Monitoring System, Lifescan, Zug, Switzerland). Composite insulin sensitivity index (CISI) was determined using the glucose and insulin values from the glucose tolerance test [27]. Serum cytokines and chemokines of the 24-week timepoint were measured using a rat 27 multiplex assay (Eotaxin, EGF, Fractalkine, IL-1α, IL-1β, IL-2, IL-4, IL-5, IL-6, IL-10, IL-12(p70), IL-13, IL-17A, IL-18, IP-10/CXCL10, GRO/KC, IFN-γ, TNF-α, G-CSF, GM-CSF, MCP-1, Leptin, LIX, MIP-1α, MIP-2, RANTES, VEGF; MILLIPLEX RECYMAG65K27PMX, Millipore, Darmstadt, Germany; assay was performed at the MultiPlex Core Facility of the University Medical Center Utrecht, The Netherlands).

### 4.3. Micro Computed Tomography (μCT)

At weeks 0, 12 and 24, μCT scans were made under anesthesia (Isofluorane—induced at 4%, maintained at 2.5%) using a Quantum FX m-CT scanner (PerkinElmer, Waltham, MA, USA). Both hind limbs were fixated in extension, and knee joints were scanned for 3 min at an isotropic voxel size of 42 μm, voltage of 90 kV, current of 180 μA, and a field of view of 21 mm. ImageJ software (ImageJ, 1.47 v, National Institutes of Health, Bethesda, MD, USA) was used for analyses. In serial 2D images in coronal orientation, the number of osteophytes and bone cysts were counted. Subchondral plate porosity was analysed by measuring the number of full and partial subchondral plate perforations. Additionally, the bone was segmented using a local threshold algorithm (ImageJ, Bernsen, using radius 5) to evaluate tibial subchondral plate thickness (μm), trabecular bone thickness (μm) and volume fraction [28]. The trabecular bone volume fraction was calculated by the ratio of trabecular bone volume (BV, in mm^3^) and total endocortical tissue volume (TV, in mm^3^). Regions of interest were manually drawn in coronal orientation in a minimum of 90 slides, starting at the back of the joint at the point where the medial and lateral compartment of the tibial epiphysis are first connected moving to the front of the joint.

### 4.4. Histology

Knee joints were harvested and fixated in 4% neutral buffered formaldehyde for 1 week and then decalcified in 0.5 M EDTA set to pH 7.0 with NaOH for 6 weeks. Every week, the samples were re-fixated in 4% neutral buffered formaldehyde for 24 h. The decalcified tissue was dehydrated in a series of ethanol (70–100% ethanol), cleared in xylene, and paraffin infiltrated. Knee joints were paraffin-embedded in a 90° angle with the patella facing down, and then coronal 5 µm sections were made at 200 μm intervals, as described in Gerwin et al. [29]. Sections were stained with Weigert’s Hematoxylin, Fast Green and Safranin-O, and the joint degeneration was then evaluated using the OARSI histopathology score for rats [29]. From each joint, the three sections that looked most damaged were scored. All sections were scored in a blinded and randomized order. The total OARSI score (0–73) is presented as the sum of the following subscores: cartilage degeneration (0–60), calcified cartilage and subchondral bone damage (0–5), osteophyte size (0–4) and synovial membrane inflammation (0–4). The cartilage degeneration score comprised the sum of four cartilage compartments (tibia and femur, medial and lateral), where each compartment was scored on a scale of 0–15. On the femur, the surgically applied grooves were not taken into account during the scoring; only the cartilage direct adjacent to the grooves was included. In addition to the scores, the tibial cartilage matrix loss width, total tibial cartilage degeneration width and significant tibial cartilage degeneration were measured, and results are expressed as a % of the total load-bearing tibial cartilage length (as described previously by Gerwin et al. [29] criteria #1, #3 and #4, respectively). Picrosirius red-stained sections were used to assess the amount of fibrosis in the infrapatellar fat pad, using three sections per knee. The percentage picrosirius red-stained area was quantified in ImageJ, in 8-bit images, using the threshold function to exclude background staining.

### 4.5. Statistics

Sample size calculations were performed in G *power 3.1.9 (Heinrich Heine University Düsseldorf, Düsseldorf, Germany), using α = 0.05 and β = 0.2. A mean difference of 3 points in the total OARSI score between the two rat strains was considered to be a clinically relevant difference. The standard deviation of the total OARSI score in previous studies using this model was 1.1 [14], resulting in an effect size d = 2.73 and a sample size of *n* = 4 per group. We included one extra animal per group to compensate for potential loss of animals, resulting in *n* = 5 rats per group. Statistical analysis was performed using Prism (v7.04, GraphPad Software, La Jolla, CA, USA) and IBM SPSS software (v25.0, IBM SPSS Inc., Chicago, IL, USA). Normality was tested with Shapiro–Wilk’s W test and checked graphically using histograms. Outcomes followed a normal distribution, and differences between two groups were tested using a two-tailed unpaired t-test when comparing between strains or using a two-tailed paired t-test when comparing within the same strain. When comparing more than 2 means, a one-way ANOVA using Sidak’s multiple comparisons test was used. Longitudinal data were tested using a mixed model (REML) analysis, with Tukey’s correction for multiple comparisons. The time point and rat strain were used as fixed effects and the individual rats as random effects. *p*-values ≤0.05 were considered statistically significant. Graphs represent mean ± standard deviation (SD), *p*-values in graphs are reported with asterisks where *p* ≤ 0.05 is *, *p* ≤ 0.01 is **, *p* ≤ 0.001 is ***, *p* ≤ 0.0001 is **** and *p* > 0.05 is not significant (NS).

## Data Availability

The data presented in this study are available on reasonable request from the corresponding author.

## Supplementary Materials

The following supporting information can be downloaded at: https://www.mdpi.com/article/10.3390/ijms23073725/s1.

## References

[1] Poole A.R. (2012). Osteoarthritis as a whole joint disease. HSS J..

[2] Li G., Yin J., Gao J., Cheng T.S., Pavlos N.J., Zhang C., Zheng M.H. (2013). Subchondral bone in osteoarthritis: Insight into risk factors and microstructural changes. Arthritis Res. Ther..

[3] Burr D.B., Gallant M.A. (2012). Bone remodelling in osteoarthritis. Nat. Rev. Rheumatol..

[4] Botter S.M., van Osch G.J., Waarsing J.H., van der Linden J.C., Verhaar J.A., Pols H.A., van Leeuwen J.P., Weinans H. (2008). Cartilage damage pattern in relation to subchondral plate thickness in a collagenase-induced model of osteoarthritis. Osteoarthr. Cartil..

[5] Botter S.M., van Osch G.J., Clockaerts S., Waarsing J.H., Weinans H., van Leeuwen J.P. (2011). Osteoarthritis induction leads to early and temporal subchondral plate porosity in the tibial plateau of mice: An in vivo microfocal computed tomography study. Arthritis Rheum..

[6] Robinson W.H., Lepus C.M., Wang Q., Raghu H., Mao R., Lindstrom T.M., Sokolove J. (2016). Low-grade inflammation as a key mediator of the pathogenesis of osteoarthritis. Nat. Rev. Rheumatol..

[7] de Visser H.M., Korthagen N.M., Muller C., Ramakers R.M., Krijger G.C., Lafeber F., Beekman F.J., Mastbergen S.C., Weinans H. (2018). Imaging of Folate Receptor Expressing Macrophages in the Rat Groove Model of Osteoarthritis: Using a New DOTA-Folate Conjugate. Cartilage.

[8] Siebelt M., Korthagen N., Wei W., Groen H., Bastiaansen-Jenniskens Y., Muller C., Waarsing J.H., de Jong M., Weinans H. (2015). Triamcinolone acetonide activates an anti-inflammatory and folate receptor-positive macrophage that prevents osteophytosis in vivo. Arthritis Res. Ther..

[9] Ioan-Facsinay A., Kloppenburg M. (2017). Osteoarthritis: Inflammation and fibrosis in adipose tissue of osteoarthritic joints. Nat. Rev. Rheumatol..

[10] Warmink K., Kozijn A.E., Bobeldijk I., Stoop R., Weinans H., Korthagen N.M. (2020). High-fat feeding primes the mouse knee joint to develop osteoarthritis and pathologic infrapatellar fat pad changes after surgically induced injury. Osteoarthr. Cartil..

[11] Courties A., Berenbaum F., Sellam J. (2019). The Phenotypic Approach to Osteoarthritis: A Look at Metabolic Syndrome-Associated Osteoarthritis. Jt. Bone Spine.

[12] Berenbaum F., Eymard F., Houard X. (2013). Osteoarthritis, inflammation and obesity. Curr. Opin. Rheumatol..

[13] Gabay O., Berenbaum F. (2009). Adipokines in arthritis: New kids on the block. Curr. Rheumatol. Rev..

[14] de Visser H.M., Mastbergen S.C., Kozijn A.E., Coeleveld K., Pouran B., van Rijen M.H., Lafeber F., Weinans H. (2018). Metabolic dysregulation accelerates injury-induced joint degeneration, driven by local inflammation; an in vivo rat study. J. Orthop. Res..

[15] Kozijn A.E., Gierman L.M., van der Ham F., Mulder P., Morrison M.C., Kuhnast S., van der Heijden R.A., Stavro P.M., van Koppen A., Pieterman E.J. (2018). Variable cartilage degradation in mice with diet-induced metabolic dysfunction: Food for thought. Osteoarthr. Cartil..

[16] Hans J.H. (2000). The Laboratory Rat, Chapter 1—History, Strains and Models.

[17] Kuhn E.R., Bellon K., Huybrechts L., Heyns W. (1983). Endocrine differences between the Wistar and Sprague-Dawley laboratory rat: Influence of cold adaptation. Horm. Metab. Res..

[18] Marques C., Meireles M., Norberto S., Leite J., Freitas J., Pestana D., Faria A., Calhau C. (2016). High-fat diet-induced obesity Rat model: A comparison between Wistar and Sprague-Dawley Rat. Adipocyte.

[19] Taylor I., Mowat V. (2020). Comparison of longevity and common tumor profiles between Sprague-Dawley and Han Wistar rats. J. Toxicol. Pathol..

[20] Fang J., Yang L., Zhang R., Zhu X., Wang P. (2015). Are there differences between Sprague-Dawley and Wistar rats in long-term effects of ovariectomy as a model for postmenopausal osteoporosis?. Int. J. Clin. Exp. Pathol..

[21] Rios J.L., Bomhof M.R., Reimer R.A., Hart D.A., Collins K.H., Herzog W. (2019). Protective effect of prebiotic and exercise intervention on knee health in a rat model of diet-induced obesity. Sci. Rep..

[22] Tellegen A.R., Rudnik-Jansen I., Pouran B., de Visser H.M., Weinans H.H., Thomas R.E., Kik M.J.L., Grinwis G.C.M., Thies J.C., Woike N. (2018). Controlled release of celecoxib inhibits inflammation, bone cysts and osteophyte formation in a preclinical model of osteoarthritis. Drug Deliv..

[23] van Lent P.L., Blom A.B., van der Kraan P., Holthuysen A.E., Vitters E., van Rooijen N., Smeets R.L., Nabbe K.C., van den Berg W.B. (2004). Crucial role of synovial lining macrophages in the promotion of transforming growth factor beta-mediated osteophyte formation. Arthritis Rheum..

[24] Blom A.B., van Lent P.L., Holthuysen A.E., van der Kraan P.M., Roth J., van Rooijen N., van den Berg W.B. (2004). Synovial lining macrophages mediate osteophyte formation during experimental osteoarthritis. Osteoarthr. Cartil..

[25] de Visser H.M., Weinans H., Coeleveld K., van Rijen M.H., Lafeber F.P., Mastbergen S.C. (2017). Groove model of tibia-femoral osteoarthritis in the rat. J. Orthop. Res..

[26] Matthews D.R., Hosker J.P., Rudenski A.S., Naylor B.A., Treacher D.F., Turner R.C. (1985). Homeostasis model assessment: Insulin resistance and beta-cell function from fasting plasma glucose and insulin concentrations in man. Diabetologia.

[27] Matsuda M., DeFronzo R.A. (1999). Insulin sensitivity indices obtained from oral glucose tolerance testing: Comparison with the euglycemic insulin clamp. Diabetes Care.

[28] Waarsing J.H., Day J.S., Weinans H. (2004). An improved segmentation method for in vivo microCT imaging. J. Bone Miner. Res..

[29] Gerwin N., Bendele A.M., Glasson S., Carlson C.S. (2010). The OARSI histopathology initiative—Recommendations for histological assessments of osteoarthritis in the rat. Osteoarthr. Cartil..

