# Supplementary material for: Sprague Dawley Rats Show More Severe Bone Loss, Osteophytosis and Inflammation Compared to Wistar Han Rats in a High-Fat, High-Sucrose Diet Model of Joint Damage
Source: Int J Mol Sci. 2022 Mar 28;23(7):3725. doi: 10.3390/ijms23073725 (PMC8999132; doi:10.3390/ijms23073725)
Supplement: Supplementary file 1 [file ijms-23-03725-s001.zip › ijms-1641054-supplementary.pdf]

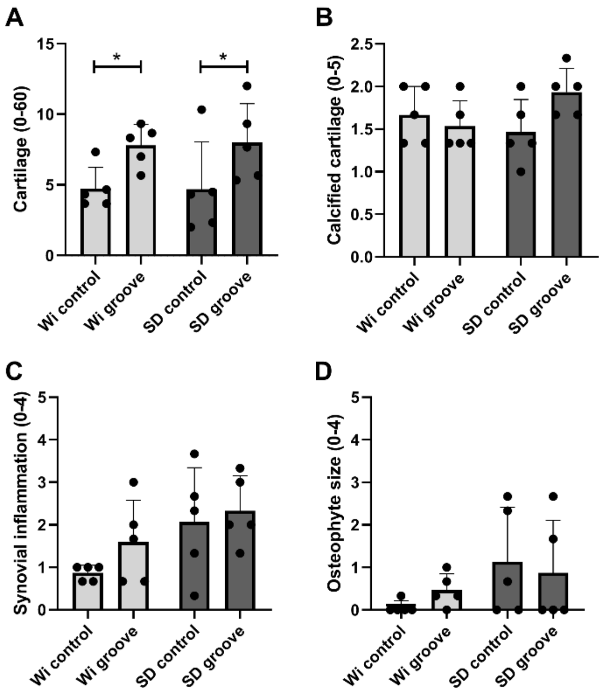

Supplementary figure S1. OARSI subscores. (**A**) Cartilage degeneration (0-60), (**B**) calcified cartilage (0–5), (**C**) synovial inflammation (0–4) and (**C**) osteophyte size (0-4). $p \leqslant 0.05$ is *.

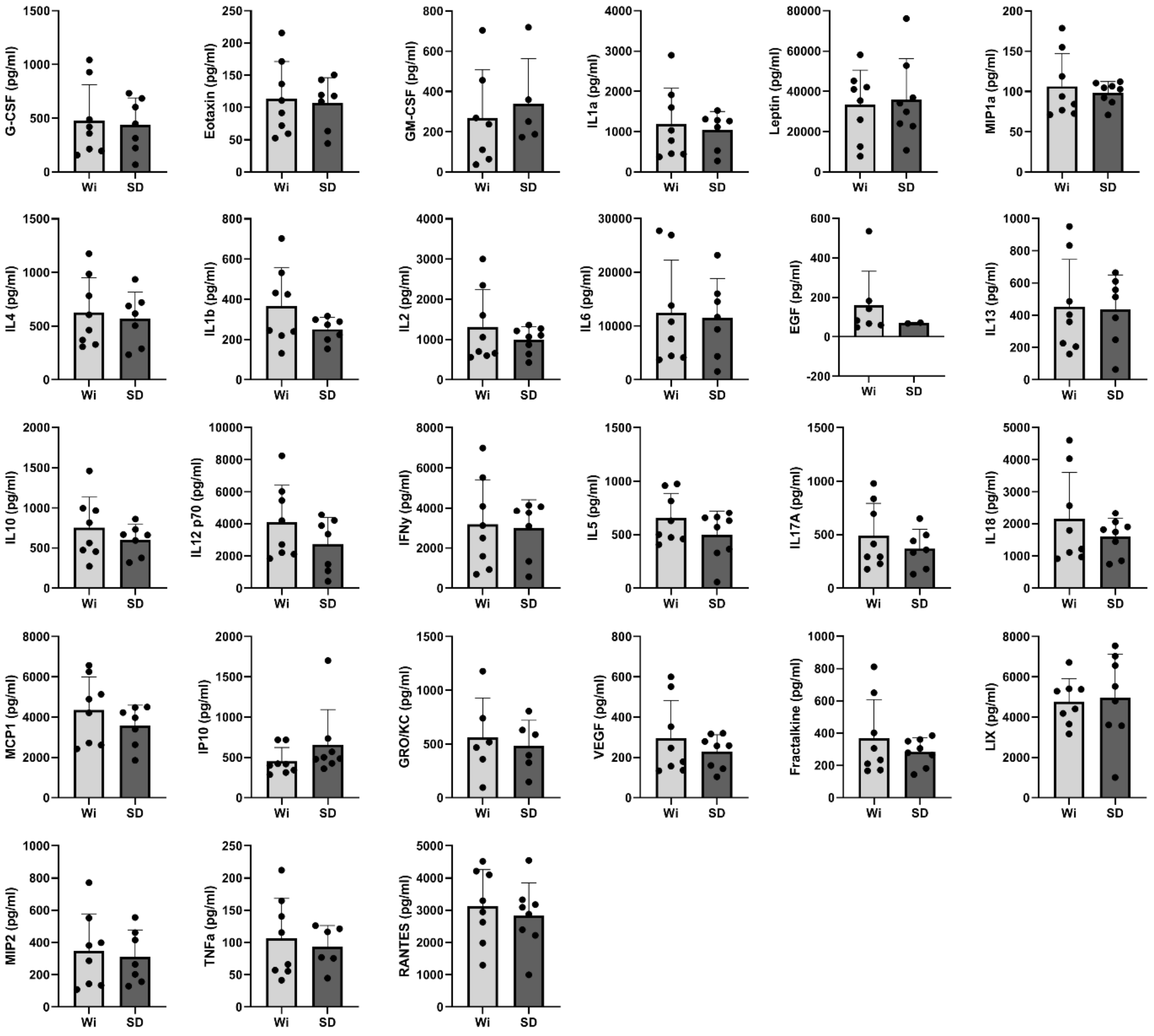

Supplementary figure S2. Inflammatory serum markers. Results of the 27 multiplex-assay showing all measured cytokines and chemokines from week 24 serum.

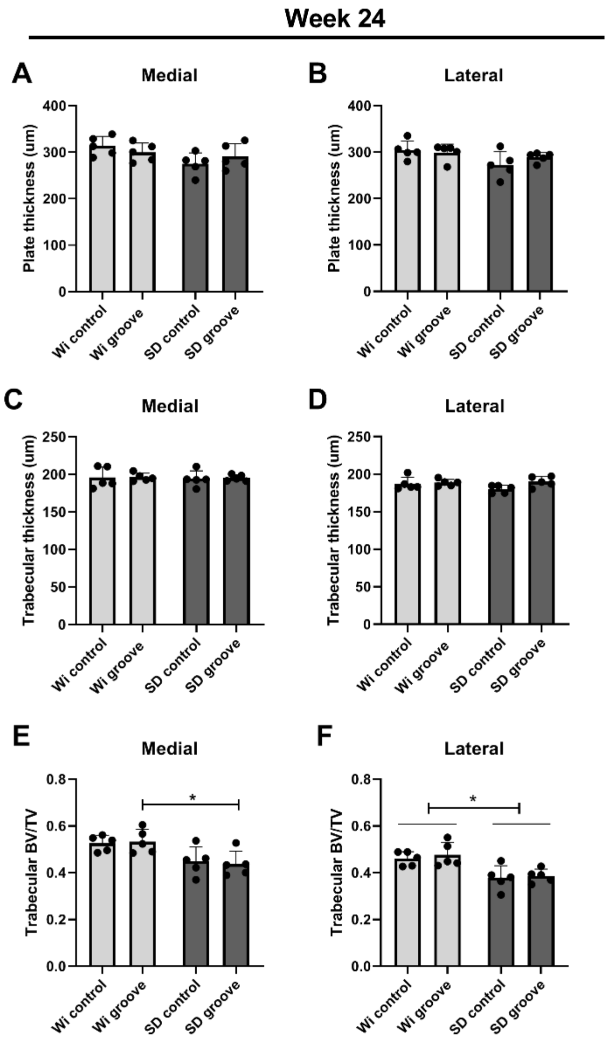

Supplementary figure S3. Absolute week 24 µCT measurements. Medial and lateral (**A,B**) tibial plate thickness, (**C,D**) tibial trabecular bone thickness and (**E,F**) tibial bone volume fraction. $p \leqslant 0.05$ is *.

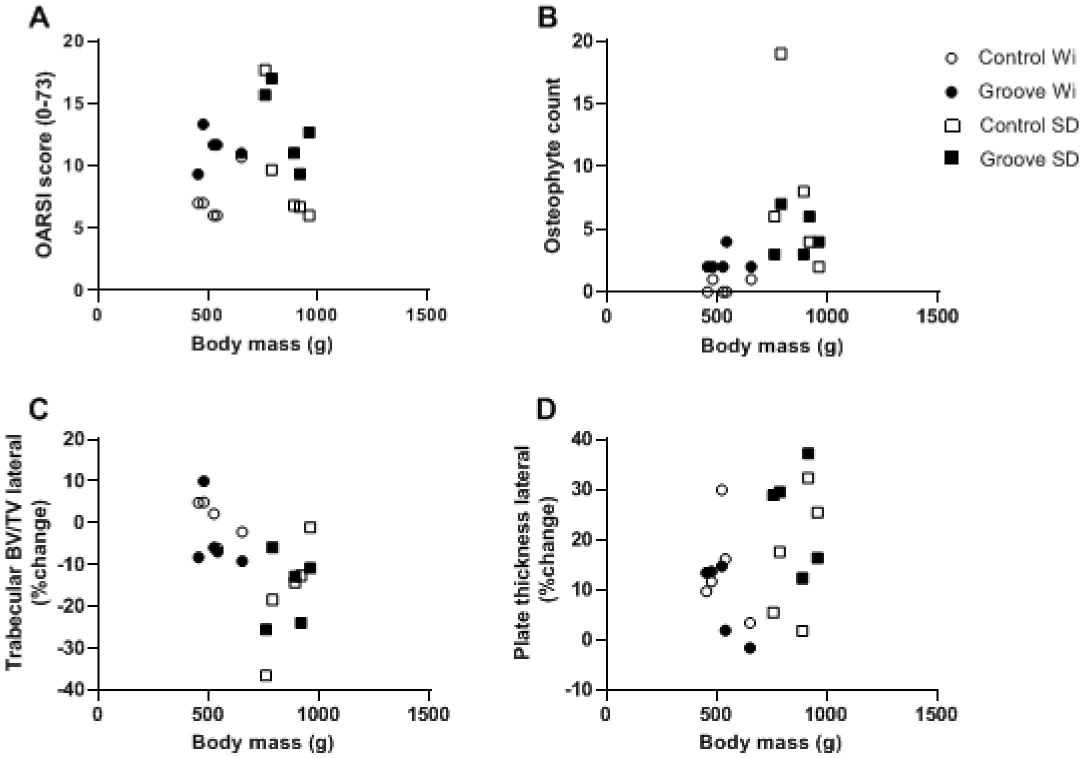

Supplementary figure S4. Body mass correlations**.** Correlation between body mass and (**A**) OARSI score, (**B**) osteophyte count, (**C**) percent change in bone volumetric fraction and (**D**) percent change in tibial plate thickness.
